# Imbalanced access to pediatric primary care in Switzerland: geographic differences and modeled future challenges

**DOI:** 10.1007/s00431-025-06441-w

**Published:** 2025-09-29

**Authors:** Michael von Rhein, Joel Hauser, Lucas Haldimann, Reto Jörg, Oliver Gruebner

**Affiliations:** 1 University Children’s Hospital Zurich, Child Development Center, University of Zurich, Lenggstrasse 30, Zurich, CH-8008 Switzerland; 2 Department of Geography, University of Zurich, Winterthurerstrasse 190, Zurich, CH-8057 Switzerland; 3 Swiss Health Observatory (Obsan), Espace de L’Europe 10, Neuchâtel, CH-2010 Switzerland; 4 Faculty of Health Sciences and Medicine, University of Lucerne, Alpenquai 4, Lucerne, CH-6005 Switzerland; 5 Centre for Primary Care and Public Health (Unisanté), University of Lausanne, Route de La Corniche 10, Lausanne, CH-1010 Switzerland

**Keywords:** Health care access, Primary care, Geographic distribution, Modeled need, Demographics, Pediatrics

## Abstract

The purpose of this study is to determine the number, age, geographic distribution, and accessibility of primary care pediatricians (PCP) in Switzerland, and to model the regional need for substitutions of soon-to-retire PCPs 5 years from now. We conducted a population-based study applying the floating catchment area method (Modified Huff-model-based Variable 3 Step Floating Catchment Area, MHV3SFCA) to calculate supply density indices (SDI) and produced accessibility maps based on data on PCP of the Federal Statistical Office (FSO) and the spatial data set of the statistics on population and households (STATPOP). In 2019, 1332 PCPs were working in pediatric practices in Switzerland (average workload: 70.4%), caring for 1,335,177 children and adolescents under the age of 15 years (i.e., 1423 per PCP). We found significant regional differences: less than 60% of the recommended PCP density, particularly in the northern part of the country and in rural regions. The needed workforce is going to accentuate markedly until 2029, when around 1/4 of Swiss pediatricians currently working in private practices will be retired. *Conclusion*: We provided a detailed analysis of the spatial distribution of PCP in Switzerland and a simulation model of the demand within the next years based on expected retirements. Our findings show current geographic disparities in pediatric primary care provision, which will accentuate and lead to a PCP shortage in many regions of Switzerland in the near future. Without sufficient compensation, adaptations of pediatric healthcare models need to be considered to avoid dramatic underservice. Our methodology is applicable to many comparable settings worldwide.

**Table:** 

**What is known:** • *pediatric care in many countries is challenged by impending retirement of older pediatric primary care providers* • *suitable statistical models are needed to identify the spatial distribution of current and future demand*
**What is new:** • *We visualized regional differences in the accessibility of paediatric primary care in Switzerland at a small-scale level, and the spatial distribution of places in need of a replacement by 2029* • *Our model is applicable to other regions, countries, and disciplines*

**Supplementary Information:**

The online version contains supplementary material available at 10.1007/s00431-025-06441-w.

## Introduction

Pediatric primary care plays a pivotal role in safeguarding the health and well-being of children, shaping their development, and preventing potential health as well as socioeconomic issues [1, 2]. In Switzerland, a central European country of high resources, pediatric primary care stands as a cornerstone in ensuring that children receive comprehensive and continuous healthcare services from infancy to adolescence [3]. With an emphasis on preventive care and early intervention, pediatric primary care in Switzerland strives to foster a healthy population, both physically and mentally [3]. The country’s approach to healthcare delivery is rooted in the principle that access to healthcare is a fundamental right for all its citizens, including children [4].

The backbone of pediatric primary care in Switzerland is a network of pediatricians in private practice. Their services include regular check-ups, vaccinations, health screenings, nutrition counseling, parental education, and mental health support, by establishing strong partnerships with parents and caregivers to enhance children’s overall health and well-being [2, 3, 5]. Government institutions play only a minor role (e.g., in the form of cantonal pediatric service for schools); clinics or similar do not fulfil a service-provision function in primary care.

As with any healthcare system, pediatric primary care in Switzerland faces its own set of challenges. These may include ensuring accessibility for all children, addressing the growing burden of chronic conditions, and adapting to changing family dynamics and healthcare needs. Research concerning geographic access to healthcare has identified critical disparities shaped by socioeconomic and demographic factors worldwide. Geographic accessibility is a significant determinant of the availability of healthcare services, with numerous studies highlighting how location plays a crucial role in both access to and the quality of healthcare received, particularly in rural settings [6].

The challenge of an ageing workforce of primary care pediatricians in private practice compounds with already existing shortages of pediatric primary care, especially in rural areas [3]. As many western countries, Switzerland has witnessed a steady rise in the average age of primary care pediatricians in private practice over the past few decades. Many of these experienced professionals are now approaching retirement age. Unfortunately, the pace of new pediatricians entering the private sector does not keep up with the number of those exiting, leading to a potential workforce shortage in the coming years [7, 8]. This demographic shift among healthcare professionals poses significant implications for the future of pediatric primary care and warrants a focused analysis of the spatial distribution of practices. As the demand for pediatric services remains steady or even increases, understanding and addressing this issue is crucial to ensure sustained access to quality care for children in Switzerland. In this context, understanding the spatial accessibility of practices is essential in developing targeted strategies to address workforce shortages.

Spatial accessibility in health care access has already been assessed for family doctors in Switzerland. The Swiss Health Observatory (Obsan) presented a new floating catchment area (FCA) method for analyzing health care density that takes into account both the availability and reachability of health care services [9–12], which is a common tool to assess spatial accessibility to healthcare. Based on the spatial distribution of the resident population and the localization of the services offered, this newly introduced method can be used to calculate a spatial accessibility index (SPAI) and a supply density index (SDI), both helping to identify regional differences in health care accessibility. Using family medicine as an example, the report described significant regional disparities, with less than 0.6 full-time equivalents (FTEs) of general practitioners per 1000 persons in the demand population in 18% of the municipalities, and 0.8 or more for 24% of the municipalities [9]. In contrast, the Swiss Medical Association (FMH) recommends a ratio of 1 FTE per 1000 inhabitants [8].

To date, a detailed analysis of the spatial distribution of primary care *pediatricians* is not available in Switzerland. Furthermore, sophisticated tools to visualize the density of available workforce in primary care and model substitution needs based on anticipated retirements are lacking. However, such knowledge and techniques would be urgently needed to find geographic disparities in pediatric primary care access. Particularly, statistical projections of the future would help to ensure equitable access to primary health care services for all children. Analyzing the spatial distribution of workforce in pediatric practices therefore becomes crucial in identifying potential gaps in access to care, particularly in rural or underserved areas. Our research questions therefore focused on investigating the number of pediatricians working in pediatric outpatient practices in Switzerland, as well as their average workload, age, and the demand from the population of children and adolescents under 15 years in 2019. We also aimed to investigate whether significant regional differences existed in the ratio between pediatricians and the local demand population. We expected an ageing population of pediatricians and an underserved pediatric population with marked regional differences. Furthermore, we aimed to examine how the anticipated loss of pediatric workforce, driven by the demographic structure of currently active pediatricians, will unfold over the coming years.

To answer the research questions, our specific goals were to (A) determine the number of pediatricians actively working in pediatric outpatient care in private practice in Switzerland and (B) explore the geographic distribution and accessibility of pediatric practices. Furthermore, we set out to (C) model the distribution of available pediatricians in private practice 10 years after baseline, based on the current demographic characteristics of active pediatricians to visualize the prognosed need of pediatricians working in private practices.

## Methods

To determine the number of pediatricians actively working in private practice in total and in relation to the demand, the structural data on medical practices and outpatient centers (MAS) of the Federal Statistical Office (FSO) were used as main data sources [13]. Because the response rate of this survey was only about 64% in 2019 [14], data of the Swiss Medical Association (FMH) were linked and added for physicians not taking part in the MAS. Analyses in this report refer exclusively to physicians with the main specializations of “pediatric and adolescent medicine” working in private practice as of December 31, 2019. For pediatricians with missing information (29%), FTEs were estimated by imputation.

To explore the geographic distribution of access to pediatric practices, we assessed health care availability and accessibility according to Jörg and Haldimann [10]. The validity of this approach has been shown by Jörg et al. [12], who applied the method in the context of general practitioners in private practice in Switzerland. Based on the method established by Jörg and Haldimann [9, 10], demand in a given region is not only measured with relation to the resident population (i.e., the population of children and adolescents under the age of 15: 1,294,918 out of 8,606,033 inhabitants in 2019 [15]), but also demand resulting from tourism and commuter flows, including cross-border commuters, is considered. Furthermore, differences in demand according to population structure and disease burden of the population (morbidity) are taken into account by means of a demand weighting using a regression model (cf. [16]). The spatial distribution of the resident population per hectare was calculated on the basis of the spatial data set of the statistics on population and households (STATPOP) of the FSO. The influence of tourism was included based on the number of overnight stays according to the accommodation statistics (HESTA) of the FSO and the billing data in the SASIS AG data pool. SASIS AG is a subsidiary of the industry association of health insurers in Switzerland and provides statistics on behalf of them. The various components are expressed in terms of equivalents of the resident population. Thus, demand can be expressed as “number of persons of the demand population.” For a more detailed description of how the demand population was derived, see Supplement A. The spatial dependencies between the service provider locations and the demand population were operationalized on the basis of travel times (travel minutes by private motorized transport calculated using ArcGIS and ESRI World Routing Service). Finally, the SDI was calculated using the MHV3SFCA method [9]. In contrast to the more commonly used SPAI, the SDI differs in its third calculation step by ignoring distances, instead summing the supply-to-demand ratios of all provider locations weighted by the respective demand probabilities, making it more interpretable as a direct supply-per-population ratio. Essentially, the SDI can be interpreted as the ratio of service provider capacity to the size of the relevant local demand population [9]. In this study, therefore, the SDI expresses the number of FTEs per 1000 children and adolescents (including demand from cross-border commuters and tourists). A more detailed description of the method and its strengths can be found in Supplement B.

To analyze the implications of the demographic structure of active pediatricians on future care, we simulated the accessibility of pediatric primary care in 2029 on the premises of no new pediatricians entering practices, empirically derived hypotheses regarding the decline in the workforce as a result of retirements and the reduction in individual working hours due to the aging of active pediatricians, and a steady demand (i.e., no population growth, no changes in request for pediatric outpatient care). The assumptions are described in more detail in Supplement C. Differences between major regions of Switzerland and rural, intermediate, and urban regions were calculated based on the definitions and typology of the Swiss Federal Statistical Office [17] and tested using ANOVA. Interaction with regional effects was assessed using a two-way ANOVA. Statistical results, including *F*-values and post hoc comparisons, are reported in Supplement D.

## Results

In 2019, 1332 physicians were working in pediatric outpatient practices in Switzerland. With an average workload of 70.4%, this corresponds to 938 FTEs. Table 1 shows the descriptive characteristics of the pediatricians. This total workforce was available to a demand population of 1,335,177 children and adolescents under the age of 15, including 1,294,918 residents as well as 32,618 and 7641 population equivalents arising from demand by tourists and cross-border commuters, respectively. Thus, an average of 1423 children and adolescents were cared for by one practitioner.

**Table 1:** Descriptive characteristics of pediatricians in private practice in 2019

Region (*n*)	Age years (SD)	Sex male, %	Work force Total FTEs
Region Ticino	50.4 (8.9)	50.1	43.5
Region Lake Geneva	48.7 (10.0)	33.3	263.4
Region Zürich	51.1 (9.5)	50.2	189.4
Central Switzerland	49.4 (9.5)	35.8	68.4
Eastern Switzerland	50.5 (9.6)	42.1	90.5
Northwestern Switzerland	51.7 (9.9)	48.4	117.8
Swiss Central Plateau	49.7 (9.6)	40.1	162.3
Total (1332)	50.1 (9.8)	42.9	937.7

Provided are *n*, %, or mean ± standard deviation

**Fig. 1:**
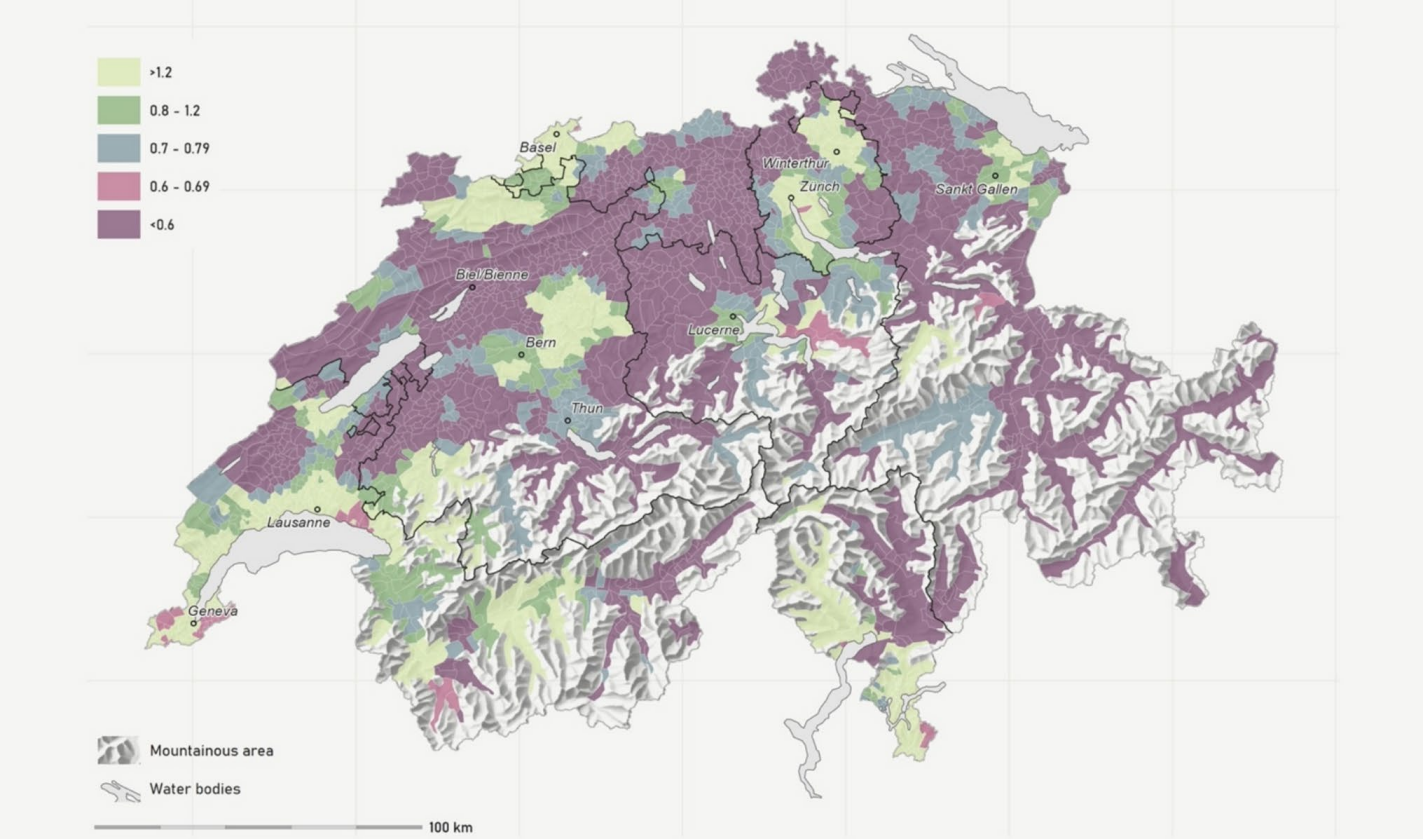
SDI map of pediatric PCPs working in private practice in 2019. Displayed are the quintiles of the supply density index (SDI) based on the Modified Huff-model-based Variable 3 Step Floating Catchment Area (MHV3SFCA) method. The SDI is first calculated per hectare and then aggregated to the municipal level using a weighted average based on demand population size. The SDI can be interpreted as the number of full-time equivalents (FTEs) per 1000 persons in the demand population. The demand population consists of the resident population under 15 years of age plus demand due to tourism and foreign countries expressed in population equivalents. Black lines: Swiss major regions. Sources: FSO - MAS, STATPOP, SOMED, HESTA; FMH - Medical Statistics; SASIS - DP, TP, ZSR; FOPH - MedReg Resolution: Municipality

Based on the SDI, the relative density of pediatricians per 100,000 children or adolescents (demand population) up to 14 years of age fluctuated between < 0.6 and > 1.2 (quintiles: see Fig. 1), with an SDI of less than 0.8 in 78.4%, and less than 0.6 in 52.9% of the municipalities. At the level of the major regions, SDI in 2019 showed significant variation, ranging from 0.52 in the north-east to 0.83 in Ticino (see Table 2 and Supplement D).

**Table 2:** Supply density differences between regions and region types (rural, intermediate, and urban)

	***n***	Mean SDI, SD		Differences	
	Municipalities	2019	2029	SDI	%
By Swiss major regions					
Lake Geneva region	480	0.76, 0.015	0.57, 0.010	−0.19	−25%
Swiss Central Plateau	674	0.57, 0.008	0.45, 0.007	−0.12	−20%
Northwestern Switzerland	300	0.52, 0.012	0.36, 0.010	−0.17	−32%
Zurich Region	162	0.64, 0.017	0.47, 0.014	−0.17	−27%
Eastern Switzerland	318	0.53, 0.011	0.40, 0.010	−0.14	−26%
Central Switzerland	162	0.58, 0.018	0.45, 0.017	−0.13	−23%
Ticino	114	0.83, 0.021	0.61, 0.018	−0.22	−26%
By region type (urban/rural typology)					
Rural	1153	0.58, 0.008	0.44, 0.006	−0.14	−25%
Intermediate	575	0.62, 0.0102	0.46, 0.009	−0.15	−25%
Urban	482	0.71, 0.0115	0.53, 0.010	−0.18	−25%
Average	2210	0.62, 0.006	0.47, 0.004	−0.15	−25%

Supply density indices (SDI) based on the Modified Huff-model-based Variable 3 Step Floating Catchment Area (MHV3SFCA) method. SDI by major region and region type are calculated as unweighted mean of SDI by municipality. The SDI can be interpreted as the number of full-time equivalents (FTEs) per 1000 persons in the demand population. Provided are mean and standard deviations (SD) in 2019, and modeled for 2029

Our analyses also showed significant differences in supply density between rural, intermediate, and urban regions, spanning from 0.58 to 0.71 in 2019 (see Table 2 and supplement D).

**Fig. 2:**
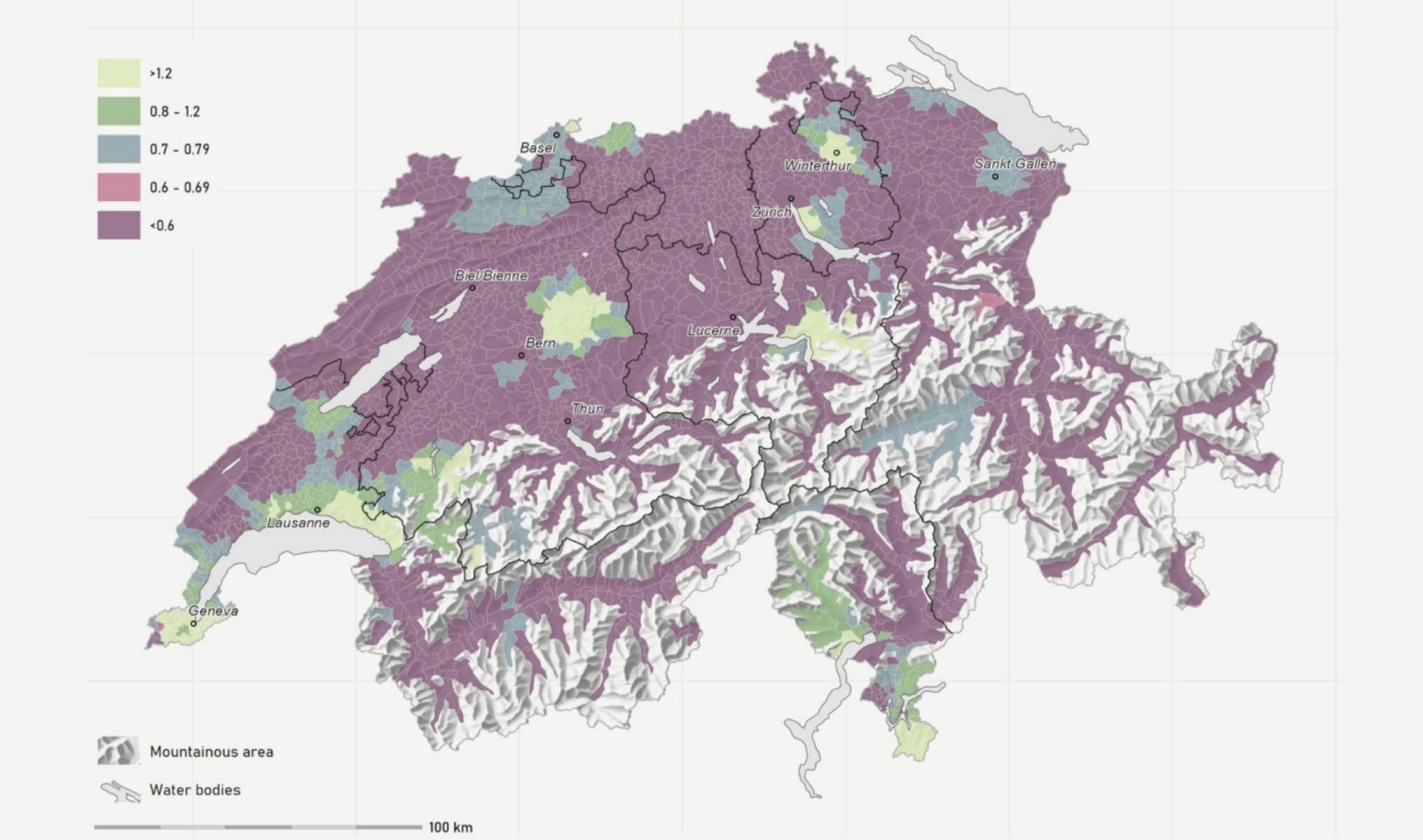
SDI map of modeled pediatric PCP workforce in 2029. Displayed are the quintiles of the supply density index (SDI) based on the Modified Huff-model-based Variable 3 Step Floating Catchment Area (MHV3SFCA) method. The SDI is first calculated per hectare and then aggregated to the municipal level using a weighted average based on demand population size. The SDI can be interpreted as the number of full-time equivalents (FTEs) per 1000 persons in the demand population. Modeled available workforce considers losses due to predicted retirement, but no compensations by successors (e.g., newly qualified or migrated specialists). The demand population consists of the resident population under 15 years of age plus demand due to tourism and foreign countries in 2019 expressed in population equivalents. Black lines: Swiss major regions. Sources: FSO - MAS, STATPOP, SOMED, HESTA; FMH - Medical Statistics; SASIS - DP, TP, ZSR; FOPH - MedReg Resolution: Municipality

To illustrate the future need for pediatricians entering the outpatient sector until 2029, we simulated an outflow based on retirement and reductions of workforce, which will sum up to an average loss of workforce by 25% until 2029. This change will affect the northwestern part of Switzerland more than the middle part of Switzerland (see Fig. 2). In absolute terms, the projected loss of workforce by 2029 has a greater impact on supply density in urban regions than in rural or intermediate ones. However, in relative terms, all regions experience an equal decline of 25%, resulting in SDI values between 0.44 and 0.53 (see Table 2).

Based on the premises of regular retirements until 2029, 237 out of 937 FTEs would be needed to maintain the level of available workforce observed in 2019. With no new pediatricians entering primary care between 2019 and 2029, the SDI would drop to less than 0.8 in 94.3%, and less than 0.6 in 74.6% of the municipalities (data not shown).

## Discussion

In this study, we estimated the number of pediatricians actively working in primary pediatric care by linking multiple datasets, calculated supply density at a small-area level, and identified significant regional disparities in access to pediatric outpatient services across Switzerland in 2019. Furthermore, we could show that in some regions, the already low baseline numbers of available pediatric primary care providers will even accentuate by the ageing community until 2029, leading to a dramatic need for substitution.

A shortage of pediatric workforce working in primary care has become a more and more pressing issue—even in high-resource countries [18–20]. In Switzerland, particularly the primary care sector has been described to face problems with too little workforce in relation to the demand population, with a trend towards a growing imbalance in the next years [8, 21]. However, many estimates are based on personal experiences, or on general numbers looking at cantons, or the country as a whole without taking regional variations into account. Our study therefore obtained reliable numbers of pediatricians working in pediatric outpatient care all over Switzerland, as well as in different regions. We found an average of 1423 children and adolescents, who were cared for by one practitioner in 2019, which is significantly more than the recommended number of 1000 children and adolescents 0–15 years of age per pediatrician [22]. To reach this goal, an additional 397 pediatricians working full time would have been needed in Switzerland already in 2019. This is reflected by the high rate of 52.9% of the municipalities with an SDI less than 0.6 in 2019, which will develop in a concerning way towards a rate of 74.6% of the municipalities with an SDI less than 0.6 until 2029 if no adequate substitution can be organized. In comparison to other high-income countries reporting pediatrician densities of around 1150 to 1700 children per doctor, Switzerland is on par with other affluent European nations, and its pediatric care capacity closely mirrors that of comparable systems [18, 23]. Nevertheless, our data clearly supports the urgent need to react politically to the imminent under-provision of pediatric primary care, particularly in rural and intermediate regions of the country.

We found marked geographic differences, with some regions exhibiting spatial density indices (SDI) around or even above 1 per 1000 in or around the larger cities, whereas more rural regions—particularly in the northern part of the country—in comparison with SDI below 0.6 were clearly underserved. Rural populations have been reported to encounter severe healthcare access barriers, including professional shortages, greater travel distances to healthcare facilities, and limited specialized services in other countries as well [6]. These barriers have been shown to correlate with poorer health outcomes and a reduced likelihood of receiving preventive care compared to their urban counterparts, necessitating focused resource allocation to enhance healthcare infrastructure in underserved areas [6]. For example, in the USA, income disparities related to geographic access were shown to be associated with significant challenges in accessing healthcare facilities, particularly in nonmetropolitan counties, leading to systemic inequities in healthcare [24]. In our study, we were able to show the applicability of our model to detect underserved regions based on publicly available data. The concept of geographic distance in healthcare accessibility has been shown to play a fundamental role in determining healthcare utilization. When health facilities are readily accessible, communities are more likely to utilize these services, reinforcing the need for strategic healthcare service planning in geographically isolated areas [25, 26]. That said, physical distance—not merely the presence of facilities—affects care management outcomes. In Switzerland, Crivelli et al. explored the decentralization of healthcare, emphasizing regional disparities in health expenditures and service accessibility across cantons [27]. Their work demonstrates that the federal structure of the Swiss health system creates inequities in healthcare delivery, often leaving rural areas underserved due to lower per-capita health expenditures and fewer available healthcare providers. Such findings indicate that improving central oversight may be beneficial in balancing these discrepancies, particularly for rural families who face unique geographic barriers [27]. Further studies showed that geographic isolation of rural communities often necessitates long travel distances to access healthcare services. Long travel distances can diminish the likelihood of individuals seeking care, as evidenced by a study by Berlin and colleagues that highlighted the relationship between rurality and the increased prevalence of avoidable hospitalizations in Switzerland [28]. Furthermore, their analysis indicated that the low density of physicians and hospital supply in these regions exacerbates the challenges faced by rural populations, leading to varied healthcare outcomes across geographic areas [28]. The regional differences found in our paper provide evidence for existing gaps in the available pediatric primary care workforce in parts of Switzerland in 2019, and a relative loss of accessibility in pediatric primary care in Switzerland since 2008 [8, 29].

The ageing pediatric workforce is expected to exacerbate the current shortage of primary care providers within the next years, causing significant imbalance between the needs of the pediatric population and the workforce, if the expected loss cannot be compensated. We found that in 2019, more than one third of all pediatricians in private practices were aged 55 years or older, thus arriving at the usual age of retirement within 10 years, leading to a marked loss of available pediatricians in private practice until 2029. This is in line with a Federal Office of Public Heath (FOPH) report from 2022, estimating the outflow of pediatric workforce in primary care to be 40% (683 FTEs) until 2030 [13]. In order to preserve the current level of available workforce, it will therefore be necessary to compensate this loss by additional efforts in pediatric resident programs, or influx of pediatricians from other countries, which in turn would weaken the available workforce in their home countries. In addition, our rather conservative model most likely underestimated the real need due to an increasing number of pediatricians working part-time [30, 31] and the growing burden of documentation and administrative duties [32].

Our results provide a far more detailed picture of the estimated need of pediatric primary care workforce than previous reports [13] and allow for a regional interpretation of the full-time equivalents (FTEs) per 1000 children and adolescents. The SDI map directly displays the number of service providers for a given demand population (FTEs per 1000 persons) and focuses on availability rather than on accessibility. The advantages of our method compared to previous models lie in the analysis of supply density independently of administrative regions. We applied the MHV3SFCA method [10], which integrates the strengths of several existing FCA methods into a single method, such as supply competition through the Huff model and the integration of variable effective catchment sizes. In addition, the MHV3SFCA method relies on the assumption of a constant overall population demand, independent of the distances between population units and supply sites, which is much more adequate in the context of health care accessibility [10]. The MHV3SFCA method also accounts for absolute difference in distances without overestimating distance effects. Using the SDI calculated based on the MHV3SFCA method, dependencies between municipalities, districts, and cantons are taken into account, which is particularly important for regions close to administrative borders. Thus, the method also allows for small-scale assessments of the availability and accessibility of health care provision (e.g., per municipality or neighborhood). Furthermore, the method incorporates not only the demand given resident population but also the demand due to tourism and commuter flows as well as regional differences of demand caused by differences in the structure of the resident population by age and sex. The method is applicable to other specialities if the necessary data linkages are approved and the relevant care provider can be delimited in the data. The challenge is not so much the applicability but rather the existing hurdles in terms of data availability and data linking procedures.

Our study has also some limitations, which are mainly based on data sources. First, our model to calculate SDI in 2029 does not take into account newly certified pediatricians. Since this figure is very volatile and difficult to predict due to the changing personal preferences of young pediatricians in terms of workload and field of work (primary care vs. specialists and practices vs. hospitals). Furthermore, it is even more difficult to predict in which regions new pediatricians would settle. Therefore, we deliberately chose only to focus on the actual total demand to be expected based on the retirement-related loss of workforce. The simulation based on these premises does not adequately predict future supply and demand. However, it is useful for identifying regions most at risk of losing supply given the demographic structure of the regional pediatrician workforce and, therefore, provides important information for research purposes as well as healthcare planning authorities. Second, family doctors or general practitioners are not included in our model, even though they play a relevant role in pediatric care (particularly in rural regions) [33]. However, these two groups of service providers are equally affected by demographic challenges [9] and clearly cannot compensate for the loss of pediatric workforce in the near future. Also, data from hospital emergency departments were not included. There has been a significant increase in the number of pediatric ED visits in Switzerland in recent years (with the exception of the SARS Cov II pandemic), which has not been considered in our analyses. For instance, pediatric ED consultations increased by an average of 30% to almost 40% in Zurich hospitals between 2017 and 2022, respectively [34, 35]. However, while pediatric EDs in hospitals have taken on increased responsibilities, they do not have the capacities to compensate for the described foreseeable gaps. Furthermore, pediatric ED service is also concentrated in larger cities, and pediatric primary care entails many aspects, which do not lie in the core competences of hospital EDs. Third, by linking various data sources, our study is the first to have a complete database of pediatricians in private practice in Switzerland. However, it is possible that the workforce is somewhat overestimated because the data sources used provide limited information on whether and to what extent individual pediatricians are clinically active in the year analyzed. In particular, there may be a delay in tracking changes during the year, so that pediatricians who quit their active service during the year are counted in the workforce. An additional link with billing data is therefore planned for future analysis so that the relevant supply of care can be determined even more precisely. For the applicability of the method itself, however, it is irrelevant how the care structure in primary care is organized, and hospital data (if available) can be included in the model without restrictions. Fourth, our study did not conduct any in-depth analyses of differences in accessibility between urban and rural areas or according to socioeconomic differences. Further studies are needed to address this issue, which could be based on the FSO’s spatial typology and the Swiss neighbourhood index of socioeconomic position (Swiss-SEP) [36, 37]. However, the results are in line with the official statements of the pediatric associations and federations in Switzerland, as well as the publications of the Federal Statistical Office and FOPH [13].

## Supplementary Information

Below is the link to the electronic supplementary material.

MOESM 1 (DOCX 56.7 KB)

## Data Availability

Our analyses are based on anonymized routine data from public statistics, which are accessible upon request. Geolocatable data on practice locations or characteristics of individual practices or doctors are subject to strict data protection regulations and were only accessible to OBSAN statisticians in the context of these analyses. Results are therefore presented in an aggregated fashion. For data protection reasons, this data cannot be made freely available.

## Declarations

### Generative AI and AI-assisted technologies in the writing process

We used *Scite*, an AI-powered research tool (www.sciete.ai) for exploring additional references for context-relevant discussion of our findings in the discussion section.
