# Supplementary material for: Imbalanced access to pediatric primary care in Switzerland: geographic differences and modeled future challenges
Source: Eur J Pediatr. 2025 Sep 29;184(10):648. doi: 10.1007/s00431-025-06441-w (PMC12477076; doi:10.1007/s00431-025-06441-w)
Supplement: Supplementary file 1 — (DOCX 56.7 KB) [file 431_2025_6441_MOESM1_ESM.docx]

**Supplement A: Operationalization of the demand population**

Based on the method established by Jörg and Haldimann [1], differences in demand were calculated 1) according to **population structure** and **disease burden of the population** (morbidity), and 2) according to **tourism** and **commuter flows**, including cross-border commuters. The demand for outpatient pediatric care was not adjusted for **commuter flows within Switzerland**, as these do not affect the regional distribution of demand for the population up to the age of 15.

For 1) we calculated the demand population as follows: The starting point is the **resident population**. The spatial distribution of the resident population per hectare can be described on the basis of the geodata set of the statistics on population and households (STATPOP) of the FSO. The **differences in demand according to population structure and disease burden** of the population (morbidity) are taken into account by means of a demand weighting. The demand weights are derived using a regression model characterizing the relationship between utilization of outpatient pediatric services and selected predictors: agegroup, gender, annual deductible (high, low), hospitalization in the previous year (yes/no), medication costs of more than CHF 5000 in the previous year (yes/no). More specifically, a Poisson-Generalized Linear Model (Poisson-GLM) was estimated using billing data of SASIS SA as data source. The Poisson-GLM method has three key advantages. First, the method always provides nonnegative predictive values for service utilization. Second, no distributional assumption is necessary with respect to the outpatient volume of services; it is simply assumed that the conditional expected value corresponds to an exponential function. Third, the method provides an unbiased estimate of the unconditional mean because the model always includes a constant. The regression model was co-developed by one of the authors in regard to a mandate from the Federal Office of Public Health (FOPH) [2]. The model's results show that utilization of outpatient services for children in the 0–5 age group is more than three times higher than those for the 11–15 age group. This seems reasonable when you consider the regular check-ups for children in the youngest age group. Based on these results we derived demand weights per age group and applied them to the population data. The implications of this weighting are limited given that the structural composition of the population up to the age of 15 does not vary greatly from region to region in Switzwerland.

Furthermore, billing data from SASIS SA was used to estimate the extent of services covered by Swiss **compulsory health insurance (OKP) for patients from abroad** (especially cross-border commuters or rather, their children) based on invoiced services provided to children residing abroad. Overall, the estimated demand for pediatric services within Swiss compulsory health insurance (OKP) for patients from abroad amounts to only 1% of the total demand, which could be considered negligible at a national level. However, it can still be a relevant factor in large centers near the border (especially Geneva and Basel), which is why it was taken into account both conceptually and quantitatively.

For 2), the number of overnight stays according to the accommodation statistics (HESTA) of the Federal Statistical Office (FSO) were used to approximate demand due to tourism, since a significant proportion of healthcare needs may be attributable to tourism, especially in tourism hotspots, another factor that can cause regional differences in healthcare demand. To quantify the demand driven by tourists, the following assumptions were made: First, it was assumed that the proportion of children (aged 0 to 15) in the tourist population would correspond to the proportion of the same age group within the Swiss resident population. Second, it was assumed that a hypothetical guest who stayed at the hotel continuously for one year (equivalent to 365 overnight stays) should be treated in the same way as a member of the resident population. Consequently, the number of overnight stays per hectare was divided by 365 days to quantify the demand generated by tourism. It could be argued that children visiting Switzerland as tourists do not have regular medical appointments. However, they are more likely to participate in leisure activities that carry a higher risk of injury, such as skiing holidays. As there are arguments on both sides, no additional adjustments were made. Overall, the estimated demand for pediatric services due to tourism amounts to only 2% of the total demand, which could be considered negligible at a national level. However, tourism can still be a relevant factor in tourist hubs such as Zermatt, which is why it was considered both conceptually and quantitatively.

**Supplement B: Calculation of the supply density index (SDI)**

To calculate the supply density index, the MHV3SFCA method [1; 3] was applied. It consists of three steps:

*Step 1*: For each combination of population $i$ and service provider location $j$, the demand probability is calculated. The demand probability ${Huff}_{ij}$ depends on the capacities of the service provider $S_{j}$ and the distance ${f(d}_{ij})$ between population and service provider as well as alternative care offers (supply competition) within the relevant catchment area of population $i$ (see the sum in the denominator):

$${Huff}_{ij}=\frac{S_{j}{f(d}_{ij})}{\sum_{j\in\left\{ d_{ij}\leq d_{i}^{rel} \right\}} S_{j}{f(d}_{ij})}\boldsymbol{I}\left( d_{ij}\leq d_{i}^{rel} \right)$$

I(.) represents an indicator function that outputs the value 1 if the condition in parentheses is true, and 0 otherwise. To approximate realistic patient behavior the relevant catchment area $d_{i}^{rel}$ is calculated separately for each population $i$ and depends on how many offers can be reached within the same distance, as well as the number of relevant supply offers Q.

$$d_{i}^{rel}=\min_{0\leq d_{r}\leq d_{max}} \left\{ d_{r} | \sum_{j} \boldsymbol{I}\left( d_{ij}\leq d_{r} \right)\geq Q \right\}$$

$Q$ represents the minimal number of supply sites $j$ which are considered as relevant options for population $i$. The maximal relevant catchment size $d_{i}^{rel}$ is derived by the minimum distance where the condition $\sum_{j} \boldsymbol{I}\left( d_{ij}\leq d_{r} \right)\geq Q$ holds true. In other words, $d_{i}^{rel}$ refers to the distance where the number of supply sites within a given distance radius $d_{r}$ is greater than or equal to the predefined threshold $Q$. The demand probability for all supply sites further away is zero. Assuming perfect patient rationality when seeking care, $Q$ should be defined as 1. This means that supply providers further away than the closest option are not considered because of greater opportunity costs related to the greater the distance. As we use distance classes, even if $Q=1$, the demand of a population is usually not attributed exclusively to the nearest service provider, but to all service providers within the corresponding radius. This means that small differences in distance within a distance class are not significant. Alternative values of $Q$ can be defined with regard of the field of analysis and the distance classes applied.

*Step 2*: Based upon the demand probabilities ${Huff}_{ij}$ calculated in step 1, a supply-ratio $R_{j}$ is determined for each supply site $j$ as follows:

$$R_{j}=\frac{S_{j}}{\sum_{i\in\left\{ d_{ij}\leq d_{max} \right\}} {Huff}_{ij}P_{i}}$$

The supply-ratio $R_{j}$ is given by the supply site capacity $S_{j}$ divided by the sum of population demand $P_{i}$ of all locations $i$ within the maximum catchment size $d_{max}$ multiplied by their probability ${Huff}_{ij}$ to seek service from supply $j$.

*Step 3*: The supply density index (SDI) is calculated as follows (Jörg & Haldimann, 2022):

$${SDI}_{i}=\sum_{j\in\left\{ d_{ij}\leq d_{max} \right\}} {{Huff}_{ij} R}_{j} 1000$$

Specifically, the SDI is the sum of the supply-demand ratios $R_{j}$of all service provider locations weighted by the respective demand probability ${Huff}_{ij}$. For scaling purposes, a multiplier of 1000 is also applied. As a result, the SDI can be interpreted directly as a ratio of FTEs per 1,000 persons in the demand population, analogously to simple patient provider ratios (PPRs). However, unlike PPRs, the SDI takes into account interdependencies between regions using the Huff model in step 1.

The MHV3SFCA method combines the strengths of previous FCA approaches and adds some new features: Briefly, the MHV3SFCA method integrates the advantages of the (E)3SFCA methods [3-6] by including supply competition through the huff probability, accounts for absolute differences in distances like the M2SFCA [7] and iFCA method [8] but does it without overestimating distance effects, incorporates variable effective catchment sizes like proposed in the iFCA [8] method, and is based on the assumption of an overall population demand that is independent of reachability. The advantages MHV3SFCA method compared to previous FCA-approaches have been demonstrated based on a simulation study [3]. Furthermore, the validity of this approach has been shown by Jörg et al. [9], who applied the method in the context of primary care in Switzerland.

For the application of the MHV3SFCA method in this study, distances were operationalized using five drive-time radiuses of 5, 10, 20, 30 and 60 minutes, and a minimal number of relevant supply sites Q was set to 1. Each travel time was assigned a distance weight based on a Gaussian distance decay function [1]. The smaller the distance, the greater the distance weight, and thus the greater the likelihood that people from the given demand population will use services from the corresponding service provider.

**Supplement C: Simulation of hypothetical supply density index (SDI) in 2029**

In order to model the distribution and accessibility of pediatricians in private practice in 2029, we calculated a hypothetical SDI based on the following assumptions: First, for the sake of simplicity, we assumed constant demand. Second, we assumed that no new pediatricians would enter practices. Third, we calculated workforce losses based on the current age of physicians as well as assumptions regarding reductions in FTEs and retirement informed by survey data collected in a study of the physician workforce in the Canton of Bern [10]. Specifically, it was assumed that the FTEs would be reduced by 50% after the age of 65, and then by a further 5% of the baseline value each year thereafter. These assumptions reflect the fact that, in the aforementioned survey, around half of physicians aged 60–64 stated that they did not plan to continue working in a clinical role five years later. However, they also reflect the fact that many physicians continue to work beyond retirement age [11].**Supplement D: Statistical testing of regional and urban-rural differences in pediatric supply density (SDI)**

To statistically assess differences in spatial accessibility to pediatric care across Swiss regions and region types, we conducted one-way and two-way ANOVAs using the supply density index (SDI) as the dependent variable. The analyses were based on the data of 2019.

1. **Regional Differences (Gross Regions)**

A one-way ANOVA revealed significant differences in SDI scores across the seven major Swiss regions (Grossregionen) (F(6, 2203) = 63.89, *p* < 0.001). Post-hoc pairwise t-tests with Bonferroni correction confirmed that the *Ticino*, *Genferseeregion*, and *Zürich* regions had significantly higher SDI values compared to most other regions (*p* < 0.001), while *Nordwestschweiz*, *Ostschweiz*, and *Espace Mittelland* exhibited significantly lower SDI scores.

1. **Urban–Rural Differences**

The SDI also varied significantly by urban–rural typology (F(2, 2207) = 42.52, *p* < 0.001). Post-hoc comparisons showed that urban areas had significantly higher accessibility than rural and intermediate areas (all pairwise comparisons: *p* < 0.001).

1. **Interaction of Region and Settlement Type**

A two-way ANOVA including both major region (Grossregion) and region type (urban–rural typology) indicated significant main effects of both factors as well as a statistically significant interaction (F(12, 2189) = 5.07, *p* < 0.001). This suggests that urban–rural differences in accessibility are not consistent across regions. For example, the urban–rural gap was more pronounced in some regions (e.g., Nordwestschweiz) than in others (e.g., Zentralschweiz).

**References**

1. Jörg R, Haldimann L (2022) Regionale Unterschiede im Zugang zur medizinischen Versorgung: Methodik zur Analyse der Versorgungsdichte und Anwendung am Beispiel der Hausartzmedizin. Obsan Bericht. OBSAN, Neuchâtel https://www.obsan.admin.ch/de/publikationen/2022-regionale-unterschiede-im-zugang-zur-medizinischen-versorgung

2. Jörg R KB, Burla L, et al. (2022) Regionale Versorgungsgrade pro Fachgebiet als Grundlage für die Höchstzahlen in der ambulanten ärztlichen Versorgung: Schlussbericht des Schweizerischen Gesundheitsobservatoriums (Obsan) und von BSS Volkswirtschaftliche Beratung im Auftrag des Bundesamts für Gesundheit (BAG). Obsan Bericht. OBSAN, Neuchâtel

3. Jörg R, Haldimann L (2023) MHV3SFCA: A new measure to capture the spatial accessibility of health care systems. Health Place 79:102974. <https://doi.org/10.1016/j.healthplace.2023.102974>

4. Wan N, Zou B, Sternberg T (2012) A three-step floating catchment area method for analyzing spatial access to health services. International Journal of Geographical Information Science 26:1073-1089. <https://doi.org/10.1080/13658816.2011.624987>

5. Luo J (2014) Integrating the Huff Model and Floating Catchment Area Methods to Analyze Spatial Access to Healthcare Services. Transactions in GIS 18:436-448. <https://doi.org/10.1111/tgis.12096>

6. Luo J (2016) Analyzing Potential Spatial Access to Primary Care Services with an Enhanced Floating Catchment Area Method. Cartographica 51:12-24. <https://doi.org/10.3138/cart.51.1.3230>

7. Delamater PL (2013) Spatial accessibility in suboptimally configured health care systems: A modified two-step floating catchment area (M2SFCA) metric. Health & Place 24:30-43. <https://doi.org/10.1016/j.healthplace.2013.07.012>

8. Bauer J, Groneberg DA (2016) Measuring Spatial Accessibility of Health Care Providers – Introduction of a Variable Distance Decay Function within the Floating Catchment Area (FCA) Method. PLOS ONE 11:e0159148. ttps://doi.org/10.1371/journal.pone.0159148

9. Jörg R HL, Rozsnyai Z, et al. (2023) Hausarztversorgung im Kanton Bern: Regionale Unterschiede im Zugang zur Hausarztversorgung. Obsan Bulletin. Schweizerisches Gesundheitsobservatorium (Obsan), Neuchâtel

10. Stierli R, Rozsnyai Z, Felber R, Jörg R, Kraft E, Exadaktylos AK, Streit S (2021) Primary Care Physician Workforce 2020 to 2025 - a cross-sectional study for the Canton of Bern. Swiss Med Wkly 151:w30024. <https://doi.org/10.4414/SMW.2021.w30024>

11. Hostettler S, Kraft E (2025) FMH-Ärztestatistik 2024 – tiefe Grundversorgerdichte. Schweizerische Ärztezeitung, Bulletin des Médecins Suisses, Bollettino dei Medici Svizzeri, <https://www.fmh.ch/files/pdf32/a250542_00_fmh_saz_11-12-2025_aerztestatistik_de.pdf:9-15>.
